# Study of informal reasoning in judicial agents in sexual aggression cases

**DOI:** 10.3389/fpsyg.2022.866145

**Published:** 2022-08-03

**Authors:** Xaviera Camplá, Yurena Gancedo, Jéssica Sanmarco, Álvaro Montes, Mercedes Novo

**Affiliations:** Unidad de Psicología Forense, Facultad de Psicología, Universidad de Santiago de Compostela, Santiago, Spain

**Keywords:** cognitive biases, motivational biases, judgment making, myths about sexual aggression, formal reasoning, informal reasoning

## Abstract

**Background/Objective:**

Judicial decisions must rest on formal reasoning. Nevertheless, informal reasoning sources (cognitive and motivational biases) were observed in judicial judgment making. Literature has identified sexual aggression cases as the most favorable for informal reasoning. Thus, a field study was designed with the aim of assessing the incidence and effects of cognitive and motivational biases in judicial agents in a case to rape to a woman.

**Methods:**

As for this, Chilean judicial agents (*N* = 217) assessed an allegation (weak evidence) of sexual assault in a case where the perpetrator was known or unknown to the victim. The judicial agents answered to a measure of the myths about sexual aggression, the attribution of responsibility to complainant, the attribution of responsibility to accused, the attribution of credibility to the complainant testimony, the attribution of a nature of a rape to the alleged facts and an estimation of the probability of false/unfounded accusations.

**Results:**

The results revealed an estimation of false/unfounded accusations of sexual aggression significantly higher than the mean of the best estimates, but into the upper limit of the best estimates; that the studied population did not share, in general, the myths about sexual aggression; and that the sources of attributional biases were driven in favor and against the complainant. Nevertheless, the case study showed that a large number of judicial agents participated of an overestimation of the probabilities of false or unfounded allegations, and of the myths about sexual aggressions and of attributional biases against the complainant.

**Conclusion:**

In conclusion, informal reasoning sources were observed in judicial agents when only formal reasoning should prevail. Thus, judicial agents should be trained to control these sources of bias substituting them by formal reasoning (evidence).

## Introduction

The literature has theorized and highlighted the impact of cognitive and motivational biases on judgment making (Kruglanski and Azjen, 1983; Montibeller and von Winterfeldt, 2015). Cognitive biases are due to the limitations of the human being to process all the information, which lead to direct attention toward certain information and discard other that could be equally relevant (Nisbett and Ross, 1980). In sum, cognitive biases arise from limitations in human information-processing substituting an expected formal reasoning (for a review see Kruglanski and Azjen, 1983). Thus, in contexts in which judgment making must rest on formal reasoning, such as legal judgments, these biases should not have a place. However, research on sexual violence has shown that judgment making about it is influenced by myths about sexual assault (Lonsway and Fitzgerald, 1994), which serve as descriptive or prescriptive cognitive tools about the causes, contexts and consequences of sexual assaults, as well as perpetrators, victims and their interaction. These types of cognitive schemes allow access to heuristic representations of information to judgment making about sexual violence (McKimmie et al., 2020). Thus, individuals sharing these myths use them to deny, minimize, overgeneralize or justify the violence of men against women (Gerger et al., 2007), while favoring risky sexual behaviors (Álvarez-Muelas et al., 2020), and a different evaluation of the same behaviors by men and women (sexual double standard; Álvarez-Muelas et al., 2021), a contingency with a high prevalence in the Hispanic context (Martínez-Gómez et al., 2021). Conversely, the perception of the complainant as chaste, respectable or sober i.e., gender victim stereotypes (counter-myths) is related to the opposite trend (Schuller et al., 2010). For this purpose, judicial judgment makers, in line with the judicial reasoning (law of precedent), are impelled to use the assignment or not of credibility (reliability in scientific judgment making models; Kaplan et al., 1978) to the testimony of the complainant (Arce et al., 2000; Du Mont et al., 2003; Page, 2007, 2010; Schuller et al., 2010; Anders and Christopher, 2011; Hine and Murphy, 2017).

On the other hand, motivational biases are characterized by a tendency to form and hold beliefs that fulfill the needs of the individual or overestimate the perceived degree of controllability of the environment (Novo and Seijo, 2010). Among the motivational biases, attributional biases have been related (Burger, 1981), which are used as means for judgment making through the attribution of responsibility or credibility (judicial task). In judgment making about sexual assault, expectations persist about how a real victim of a sexual assault behaves, which are a breeding ground for attributional biases (Smith and Skinner, 2017) that are not present in other types of crimes not including the issue of consent that plays a critical role in rape cases (Bieneck and Krahé, 2011). Thus, these sources of bias are used to attribute responsibility to the complainant and aggressor, to attribute credibility to the testimony of the complainant (proof of charge, while the testimony of the accused is not evidence of the charge, so from its evaluation cannot be derived criminal responsibility) or to attribute to the alleged facts nature of a sexual assault (most sexual assault complaints have to be resolved on whether the facts are an assault or a consensual relationship).

Biases acquire a functional nature as the evidence is weak and lose it when the evidence is strong (Visher, 1987; Kahneman, 2011; Butterfield and Bitter, 2019; Nitschke et al., 2019). In crimes committed in the private sphere (e.g., domestic violence, sexual assault) there are, therefore, few media of burden of proof beyond the testimony of the complainant and the evaluation of the damage to the complainant (Arce, 2017). Hence, trials in these crimes are conducive to the manifestation of bias.

These types of cognitive and motivational biases affect not only judicial judgment making of laypersons (the vast majority of research has been carried out with jurors, i.e., laypersons; Schuller et al., 2010), but also of law professionals (Fitzmaurice and Pease, 1986; Saks and Kidd, 1986; Fariña et al., 2002; Arce et al., 2003). In the European and Anglo-Saxon context, the presence and impact of myths about sexual violence on police and judicial agent samples has been widely documented (Camplá et al., 2017; Smith and Skinner, 2017; Temkin et al., 2018). Nevertheless, not all have the same meaning in this type of population. Thus, one of the most prevalent myths reported in the population of professionals with procedural or judicial competences (Police, Prosecutors, Judges) is the one referring to false (deliberately fabricated)/unfounded (not deliberately fabricated, baseless, groundless) accusations, based on the belief that women allege for revenge, for profit or regret (Lisak et al., 2010; Lonsway, 2010; Ferguson and Malouff, 2016). As well, the Chilean Law of Precedent establishes that the testimony of the complainant is not sufficient evidence if there is some benefit, revenge or repentance in it. Hence, if this were the only proof against the accused, the judicial criterion would classify the case as evidenceless and it would be closed or archived, expanding in this group unfounded to evidenceless.

In any case, the estimation of the probability of false or unfounded accusations has been considered as a source of bias in trials in sexual assault crimes. In this regard, a meta-analytical review (Ferguson and Malouff, 2016) found a high inter-study variability and that the results were subject to the effect of moderators that could not be identified due to lack of studies (possibly moderators of the effects are the definitions of false and unfounded complaint and the type of population). While waiting to know these moderators, the lower (0.012) and upper (0.174) limits reported demarcate the estimates within normality (between the interval of the best estimates), with the lower and upper estimates being outside the normal range.

In Latin-American judicial setting, it has been argued that investigative actions and judicial judgment making in sexual assault cases may be contaminated by prejudices about sexual assault and the complainant (Salinas et al., 2015). Hence a quasi-experimental field study with Chilean judicial agents (i.e., law enforcement officers, correction officers, prosecutors, and judges), as professionals with procedural or judicial competences, to estimate the probability of false or unfounded complaints, the incidence of myths about sexual assaults, and the incidence and effects of motivational biases in judgment making was designed.

## Materials and methods

### Participants

Chilean judicial agents participated in the study, of which 60 were gendarmes (correctional officers), 76 police (law enforcement officers), 67 prosecutors and 14 judges. The distribution of the participants by age, sex, seniority in the position, and specialized training in sexual crimes can be seen in Table 1.

**TABLE 1:** Socio-demographic characteristics of the participants.

Variable		Gendarmes	Polices	Prosecutors	Judges
Age		*M* (*SD*)	*M* (*SD*)	*M* (*SD*)	*M* (*SD*)
		35.7 (6.2)	41.7 (3.7)	39.2 (6.1)	51.9 (8.5)
		*n* (%)	*n* (%)	*n* (%)	*n* (%)
Sex*^a^*	Men	46(78)	70 (92.1)	28 (41.8)	4 (28.6)
	Women	13 (22)	6 (7.9)	39 (58.2)	10 (71.4)
Training in sexual crimes		2 (3.4)	12 (15.8)	35 (52.2)	11 (78.6)
Seniority in the position	<10 years	8 (13.3)	0 (0)	35 (52.2)	0 (0)
	>10 years	52 (86.7)	76 (100)	32 (47.8)	14 (100)
Total		60	76	67	14

M(SD), Mean(Standard Deviation); n(%), Number of participants(observed percentage).

*^a^*1missing value.

### Procedure and design

A quasi-experimental field study was designed. Firstly, the Gendarmerie, Investigative Police, National Prosecutor’s Office and Judicial Power headquarters were required to authorize the data collection among their members, presenting the investigation design and measures. Once approval was obtained, participants were contacted by their headquarters asking for voluntary participation. Those who agreed to participate were contacted personally by researchers, signed an informed consent, and endorsed the measures. In compliance with Chilean regulations, the ethical principles of beneficence, autonomy, and justice were respected. Data collection was individual and, once the sociodemographic information was obtained, to counterbalance the interaction between measures, the order in which the measures were obtained was rotated (standard rotation procedure), that is, A, B, C,… F; B, C,…. A;… Data were collected individually from July 2018 to February 2019.

### Measure instruments

A sociodemographic questionnaire was created in which the participants reported age, sex, length of service (< 10 and > 10 years, which is the criterion with which judicial agents are considered to be highly experienced public officials) and having completed specialized training in sexual crimes in his/her position (yes vs. no). Additionally, they were asked to estimate the percentage (from 0 to 100), i.e., probability of false/unfounded accusations regarding sexual crimes according to their own experience.

For the measurement of myths about sexual aggression, it was applied the Chilean adaptation (Camplá et al., 2019a,b) of the AMMSA Scale (Acceptance of Modern Myths about Sexual Aggression; Gerger et al., 2007). This adaptation, with a unidimensional structure, consists of 14 items to which people respond on a 7-point Likert-type scale from *completely disagree* (1) to *completely agree* (7). With study participants, the scale showed excellent internal consistency, α = 0.907.

For the evaluation of attributional biases in the study population, 2 vignettes (± 300 words) with weak evidence (only the accusatory testimony of the complainant) about a rape allegation were developed. The difference between the two scenarios was that the accused was or was not known to the complainant. Previously, a group of 10 researchers with experience in research design and with knowledge of psychology and law evaluated the incriminating evidence in the scenarios on an 11-point scale (Thurstone’ procedure) if the charging evidence was weak (1, *extremely weak*) or strong (11, *extremely strong*). The results showed a *Mdn* = 1, Mode = 1, max. score = 3, Q1 = 1, Q2 = 2, and IQR = 1. Thus, the scenarios were assessed for the evaluators as weak evidence. Participants answered to a validated measure of the attributional biases (Arce et al., 2003), consisting in 5 questions:

(1) To what extent do you attribute responsibility to the complainant in the reported facts? Where: 0 = *Not at all responsible*; 1 = *Slightly responsible;* 2 = *Somewhat responsible*; 3 = *Mostly responsible*; 4 = *Completely responsible*.

(2) With what probability do you attribute the complainant’s ability to have prevented the reported incident? Where: 0 = *Not probable*; 1 = *Slightly probable*; 2 = *Somewhat probable*; 3 = *Moderately probable*; 4 = *Extremely probable*.

(3) To what extent do you attribute responsibility to the defendant in the reported facts? Where: 0 = *Not at all responsible*; 1 = *Slightly responsible*; 2 = *Somewhat responsible*; 3 = *Mostly responsible*; 4 = *Completely responsible*.

(4) To what extent do you attribute credibility to the incriminating testimony given by the complainant? Where: 0 = *Not true*; 1 = *Slightly true*; 2 = *Somewhat true*; 3 = *Moderately true*; 4 = *Completely true*.

(5) With what probability do you attribute nature of a rape to the alleged facts? Where: 0 = *Not probable*; 1 = *Slightly probable*; 2 = *Somewhat probable*; 3 = *Moderately probable*; 4 = *Extremely probable*.

These variables are measuring the same construct (α = 0.70; $\bar{r}$ = 0.244; *r*_s_ > 0.142, *p* < 0.05), the attributional bias in judgment making.

### Data analysis

Mean comparisons with a test value was computed with one sample *t*-test, being effect size estimated with Cohens’s *d* and quantifying the magnitude in terms of *r* (Redondo et al., 2019). Mean comparisons of repeated measures were processed performing a MANOVA estimating multivariate effect size in percentage of explained variance (η^2^) and bivariate effect size in Cohen’s *d* (within formula).

Observed contingencies were contrasted with a constant computing Z score for the difference between proportions. The constants were taken as follows (Fandiño et al., 2021): (a) a trivial probability (≤ 0.05, insignificant probability); (b) a common probability (= 0.5, probable, observed in 50% of the population); and (c) a normal probability (≥0.90; normal, observed in 90% or more of the population). The magnitude of the increase or decrease of the observed contingency (effect size) was valued in terms of the Effect Incremental Index (EII; Arias et al., 2020).

As to compare the observed probabilities of false or unfounded allegations into the three classification categories (lower than best estimates, within best estimates, higher than best estimates) and between subsamples, the 95% confidence interval for each observed probability was computed. If the confidence intervals overlap, then the observed probabilities are equal, meanwhile if the confidence interval do not overlap, the observed probabilities are significantly different. Equally, the 95% confidence interval of the observed mean in the attributional biases were calculated to compare means between the sources of biases. Likewise, if the confidence intervals overlap, then the observed means are equal, meanwhile if the confidence interval do not overlap, the observed means are significantly different. The lower limit of the 90% confidence interval [i.e., *M* – (1.645 * *SD*)] for the population distribution in the attributional biases was valued to know if a trivial (insignificant) effect (1 = slightly) was or not within the normal distribution.

The population distribution and mean comparison study is of great scientific relevance, but results are insufficient to whole generalization to population as the effect is not general. Thus, the estimation of the margin of error of the resulting statistical model complements the significant model and should be reported. Hence, the Probability of an Inferiority (PIS) or a Superiority (PSS) Score (Gancedo et al., 2021a), i.e., the probability of subjects of the higher mean score group obtains a lesser score than the mean of the higher group (PIS), or the probability of subjects of the lower mean score group obtains a great score than the mean of the higher group (PSS), was estimated.

## Results

### Estimation of false or unsubstantiated reports in reports of sexual assault

The mean probability (*M* = 0.200) reported by the study population (22 participants did not answer this question, *N* = 196) of false/unfounded complaints is significantly higher, *t*(195) = 10.12, *p* < 0.001, than the mean (*M* = 0.052) of the existing studies (test value from a meta-analytic review; Ferguson and Malouff, 2016) in the literature, with a large effect size, *d* = 0.98, implying a 44.0% (*r* = 0.440) of increase in the estimate over the average of the best estimate. However, given the high variability in the estimates (heterogeneity in the studies) and that the moderating variables of the effect are unknown, although it is believed that the measure of only false complaints gives rise to lower rates than when it also includes unfounded complaints, the observed mean was contrasted with the upper limit of the estimates (0.174), finding that the mean of the study population was equal to the upper limit, *t*(195) = 1.76, *ns*. Hence, the estimates of false/unsubstantiated allegations are at the upper limit of the best estimates.

Regarding the case study (see Table 1), the reported probability was recoded in three categories according to the results of the meta-analytic review by Ferguson and Malouff (2016): Lower than best estimates (estimated probability ≤ 0.012); within the best estimates (0.012 < estimated probability ≤ 0.174) and higher than best estimates (estimated probability > 0.174). The results showed, for the population of judicial agents, a non-trivial underestimation (> 0.05) of the probability of false/unfounded complaints, *Z*(*N* = 196) = 7.25, *p* < 0.001, resulting the increase over a trivial probability of 69.3% (EII = 0.693), being common (= 0.5) the overestimation (0.454), *Z*(*N* = 196) = 1.29, *ns*. On the other hand, the observed probability within the best estimates (0.383) is significantly lower than that expected for this contingency (0.90, normal probability), *Z*(*N* = 196) = –24.13, *p* < 0.001, with the decrease in 57.4% (EII = 0.574) in relation to the normal probability. Comparatively, the probability of overestimation is significantly higher than that of underestimation, χ^2^(*N* = 121) = 26.85, *p* < 0.001. The sample was divided in agents with incriminating procedural functions (e.g., investigation, apprehension, detention of individuals suspected of criminal offenses, prosecution of the defendant or dismiss the case), law enforcement officers (polices) and prosecutors, and agents without procedural functions (they do not make decisions about the process), gendarmes and judges, the results showed (see Table 2) the same contingencies (confidence intervals for the observed proportions overlap) in both subsamples in lower than best estimates and higher than best estimates.

**TABLE 2:** Contingency table of grouping estimates and population.

Estimation false/Unfounded allegations	Gendarmes/Judges *f*(p[95% CI])	Polices/Prosecutors *f*(p[95% CI])	Total *f*(p[95% CI])
Lower than best estimates (<0.012)	13 (0.169 [0.085, 0.253])	19(0.160 [0.094, 0.226])	32 (0.163 [0.111, 0.215])
Within best estimates (0.012, 0.174)	29 (0.377 [0.269, 0.485)	46 (0.383 [0.296, 0.470])	75 (0.383 [0.315, 0.451])
Higher than best estimates (>0.174)	35 (0.455 [0.344, 0.566])	54 (0.454 [0.365, 0.543])	89 (0.454 [0.384, 0.524])
Total	77	119	196

f(p[95% CI]), frequency observed probability [95% Confidence Interval].

### Study of the incidence of myths about sexual assault

The results (see Table 3) exhibited in the studied population (judicial agents) a systematic trend of disagreement with the myths about sexual assaults, except for the accusation of sexual violence to obtain custody, labeling harmless conduct as sexual harassment in the battle between the sexes; and the interpretation of harmless behaviors as sexual harassment at work, in which the degree of agreement is not positioned. As for the myths overall score (a composite score was computed), the results exhibited that judicial agents (*M* = 3.33) do not share the myths about sexual assault, *t*(217) = –8.24, *p* < 0.001, *d* = 0.56.

**TABLE 3:** One sample *t*-test for the contrast of the acceptance of the myths about sexual aggression (test value: 4, Neither agree nor disagree).

Myths about sexual assault	*M*	*t*	*d*	PSS
1. Para conseguir la custodia de sus hijos, las mujeres a menudo acusan falsamente a sus exmaridos (o exparejas) de tener inclinaciones hacia la violencia sexual [To get custody for their children, women often falsely accuse their ex-husband of a tendency toward sexual violence]	3.82	–1.57	–0.11	0.456
2. Interpretar gestos inofensivos como “acoso sexual” es un arma muy común en la batalla de los sexos [Interpreting harmless gestures as “sexual harassment” is a popular weapon in the battle of the sexes]	3.82	–1.59	–0.07	0.472
3. Mientras no vayan demasiado lejos, los comentarios e insinuaciones que se hacen a las mujeres simplemente quieren decirle que es atractiva. [As long as they don’t go too far, suggestive remarks and allusions simply tell a woman that she is attractive]	3.55	–3.73***	–0.25	0.401
4. La mayoría de las mujeres prefieren ser elogiadas por su físico que por su inteligencia [Most women prefer to be praised for their looks rather than their intelligence]	2.97	–8.28***	–0.55	0.291
5. Aunque a las mujeres les gusta hacerse las tímidas, eso no significa que no quieran sexo. Women like to play coy. This does not mean that they do not want sex.	3.01	–8.08***	–0.47	0.319
6. Muchas mujeres tienden a exagerar el problema de la violencia machista [Many women tend to exaggerate the problem of male violence]	3.59	–3.10**	–0.23	0.409
7. Cuando una mujer soltera invita a un hombre soltero a su casa, está indicando que no es reacia a mantener relaciones sexuales [When a single woman invites a single man to her flat she signals that she is not averse to having sex]	2.65	–12.56***	–0.73	0.233
8. Cuando se habla de “violación en el matrimonio,” no hay una distinción clara entre coito conyugal normal y violación [When defining “marital rape,” there is no clear-cut distinction between normal conjugal intercourse and rape]	2.74	–10.04***	–0.60	0.274
9. La sexualidad de un hombre funciona como una olla a presión; cuando la presión es muy alta. tiene que “soltar vapor”[A man’s sexuality functions like a steam boiler – when the pressure gets too high, he has to “let off steam”]	2.65	–11.11***	–0.66	0.255
10. El debate sobre el acoso sexual en el trabajo ha provocado que muchos comportamientos inofensivos sean malinterpretados como acoso sexual [The discussion about sexual harassment on the job has mainly resulted in many a harmless behavior being misinterpreted as harassment]	3.80	–1.66	–0.10	0.460
11. En las citas lo que suele esperarse es que la mujer “ponga el freno” y el hombre “siga adelante” [In dating situations, the general expectation is that the woman “hits the brakes” and the man “pushes ahead”]	2.85	–10.27***	–0.59	0.278
12. Pese a que las víctimas de robo armado corren un mayor peligro de vida, reciben mucho menos apoyo psicológico que las víctimas de violación [Although the victims of armed robbery have to fear for their lives, they receive far less psychological support than do rape victims]	4.29	2.23*	0.16	0.564
13. El alcohol es a menudo el causante de que un hombre viole a una mujer [Alcohol is often the culprit when a man rapes a woman]	3.31	–5.33***	–0.34	0.367
14. Muchas mujeres tienden a malinterpretar un gesto bienintencionado como “acoso sexual” [Many women tend to misinterpret a well-meant gesture as a “sexual assault”]	3.54	–3.90***	–0.26	0.397

df(216); *p < 0.05; **p < 0.01; ***p < 0.001.

In contrast, the population reported participating in the attributional bias of greater provision of psychological support from the community to rape victims than to victims of very violent crimes (robbery with the use of weapons).

However, the case study (see PSS in Table 3), necessary in this type of population because the decisions are individual and the biases derived from the myths are manifested individually, warns of bias rates (in agreement with the myths) in this population they range from approximately 1/4 (± 25%) for myths (see the content of the myths in Table 3) 4, 7, 8, 9, and 11; around 1/3 (± 33%) for myths 5, 13, and 14; around 1/2.5 (± 40%) for myths 3 and 6; approximately 1/2 (± 50%) for myths 1, 2, and 10; and more than 1/2 (+50%) in myth 12. In general (composite score), about 1/4 of judicial agents agree (PIS = 0.291) with myths about sexual assault.

### Evaluation of the effect of attributional biases in judgment making

The results of the study of the extra-legal evidence (see Table 4) revealed a significant effect (>1, 1 = trivial effect) of the biases of attribution of responsibility to the complainant and the accused, of truthfulness to the complainant, of a nature of rape to the alleged facts, and of a preventive role for the complainant with effect sizes greater than large (*d* > 1.20).

**TABLE 4:** One sample *t*-test of the attributional bias measures with a trivial attribution as test value (1, slightly).

Source of attributional bias	*t*	*M*	*d*
Attribution of responsibility to the complainant	34.22***	3.08	3.28
Attribution of responsibility to the accused	48.38***	3.43	4.65
Attribution of veracity to the complainant	32.48***	2.89	3.15
Attribution of nature of a rape to the facts?	57.40***	3.57	5.52
Attribution of prevention of the incident to the complainant	15.66***	2.14	1.51

*df(433)*. ***p < 0.001.

The comparison of the bias attributing responsibility to the complainant and the accused (see Table 5) showed a significantly higher attribution of responsibility or the accused (confidence intervals do not overlap, and the mean is higher for the accused). On the other hand, the bias of attribution to the complainant of the ability to prevent the incident is the one with lower incidence (the upper limit of confidence for the mean is lower than the upper limit of the other biases), while the attribution to the facts of a nature of rape, the one with higher incidence (the lower limit of the confidence interval for the mean is greater than the upper limit of the remaining biases). In an intermediate position is the attribution of veracity to the testimony of the complainant.

**TABLE 5:** Confidence interval for the observed mean and populational lower limit of the normal distribution of the extralegal evidence variables (attributional biases).

Variable	*M* [95% CI]	90% NI lower limit
Attribution of responsibility to the complainant	3.08 [2.96, 3.20]	0.99
Attribution of responsibility to the accused	3.43 [3.33, 3.53]	1.70
Attribution of veracity to the complainant	2.89 [2.80, 2.98]	0.90
Attribution of nature of a rape to the facts	3.57 [3.49, 3.65]	2.04
Attribution of prevention ability to the complainant	2.14 [2.00, 2.28]	–0.36

N = 434; M [95% CI], Mean [95% Confidence Interval for the mean]; 90% NI lower limit, 90% normal interval lower limit.

The normal interval includes the triviality (1) in the attribution of responsibility to the complainant, but not to the accused; that is, the non-attribution of responsibility to the accused is out of normality (abnormal), meanwhile triviality in the attribution of responsibility to the complainant is normal (falls into normality). It also falls within normality (90% normal interval lower limit < 1) not attributing sufficient veracity to the complainant’s testimony, as well as not attributing to the complainant the ability to prevent the incident, while not attributing a nature of rape to the facts of rape is abnormal (90% normal interval lower limit > 1). Succinctly, not attributing responsibility to the accused or not qualifying the facts described as a rape is abnormal in this population.

Performed a repeated measures MANOVA on the extralegal evidence measurement variables i.e., attributional biases, the results showed a significant multivariate effect, [*F*(5, 211) = 6.47, *p* < 0.001, 1–β = 0.997], for the perpetrator factor (between factor: known vs. unknown), which explains 13.3% of the variance. As for univariate effects (see Table 6), the results exhibited a significant higher attribution of responsivity, of veracity in the testimony and of capacity to prevent the incident to complainant for unknown perpetrators in comparison with known perpetrators. However, 34.0, 30.2, and 28.8% of the judicial agents (see PIS in Table 6) would attribute less responsibility, truthfulness in the testimony and prevention capacity to the complainant when the perpetrator is unknown (statistical model error).

**TABLE 6:** Univariate effects on the attributional biases for the perpetrator factor.

Variable	*M*_Uk_	*M*_K_	*F*	1-β	*d*	PIS
Attribution of responsibility to the complainant	3.16	3.00	4.39*	0.550	0.28	0.340
Attribution of responsibility to the accused	3.50	3.36	2.86	0.391	0.23	0.409
Attribution of veracity to the complainant	3.06	2.72	14.33***	0.965	0.52	0.302
Knowledge of the crime as such	3.62	3.52	2.95	0.401	0.24	0.405
Attribution of prevention ability to the complainant	2.35	1.94	16.84***	0.983	0.56	0.288

Within-subjects effects.

df (1, 215); M_Uk_, mean of the unknown perpetrator condition; M_K_, mean of the known perpetrator condition.

*p < 0.05; ***p < 0.001.

## Discussion

Regarding the incidence of myths about sexual assault, the results obtained with Chilean judicial agents showed, in general, a systematic tendency of disagreement with the myths about sexual assault. Bearing in mind the theoretical content categories of the scale (Gerger et al., 2007), they express disagreement with the denial of the scope of sexual violence, as well as with gender stereotypes about male sexuality, the beliefs that exonerate perpetrators of violence, and the naturalization of male coercion. At the same time, they are in favor of the demands of the victims and of the policies designed to address the effects of sexual violence. Nevertheless, there is no systematic trend of agreement or disagreement regarding the false accusation of sexual violence to obtain custody, and the interpretation of harmless gestures as sexual harassment. On the contrary, they participate in the myth of positive discrimination “receive more psychological support” rape victims compared to victims of armed robbery that are not based on the provision of greater support to victims of sexual assault. De facto, all Chilean victims of violent crimes (e.g., sexual assault, kidnapping, robbery with violence) receive legal assistance, psychological therapy and social support. In any case, the results do not endorse a generalized cognitive bias in this population contrary to those reporting sexual assault victimization, observed in other studies (Sleath and Bull, 2012; Smith and Skinner, 2012; McMillan, 2016; Hine and Murphy, 2017; Temkin et al., 2018).

However, the case study, necessary, as it deals with personal biases (De Neys and Bonnefon, 2013), indicates that a large part of the legal agents participate in the myths about sexual assaults, with agreement rates that oscillate between ± 25%, at +50%. In sum, although a systematic bias trend is not observed in the population studied, a significant and large prevalence of cases has been recorded (ranging from 1/4 to more than 50% of judicial agents, depending on the myths).

Myths are part of sources of informal reasoning (evidence is replaced by the myths), as opposed to formal reasoning (evidence based) that must support judicial judgments (Kruglanski and Azjen, 1983). In practice, myths are within the cognitive bias “preconceived ideas or theories” that predispose the individual to adopt uncertain ideas (e.g., myths) that guide judgment making *via* information processing strategies such as presumed covariation (e.g., correlation between myths and false or unfounded allegations), representativeness (e.g., overestimation of the probabilities of false or unfounded allegations related to myths) or causality (e.g., myths are the causes of false or unfounded allegations) (Fariña et al., 2002). These preconceptions maximize judgments based on myths, avoid discordant information, and lead to cognitive savings for the individual (Ross, 1977).

In relation to the estimation of false or unfounded reports (representativeness cognitive bias), it was found that the mean probability reported by the study population is significantly higher than the mean reported in the literature (0.052; Ferguson and Malouff, 2016), and is in the upper limit of the best estimates. In addition, the study of cases, according to the results of the meta-analytical review by Ferguson and Malouff (2016), has allowed in order to establish for legal agents a common overestimation of false or unfounded complaints, as well as a non-trivial underestimation, although the overestimation is significantly greater than the underestimation (Venema, 2016). In this sense, research has shown that high estimates are related to a lower allocation of credibility and receptivity toward those who report (Lonsway et al., 2009; Mennicke et al., 2014). Likewise, judicial agents with differentiated procedural functions (who activate the search for evidence or file the process), police and prosecutors, and operators without procedural functions (they do not make decisions about the process), gendarmes and judges, did not reveal differences in the estimation of false or unfounded complaints, which may condition the decision about a criminal prosecution or contribute to poor investigations (Mennicke et al., 2014; Hohl and Stanko, 2015; O’Neal et al., 2015; Carboné-López et al., 2016). In any case, it is remarkable that the assessment of the probability of false or unfounded complaints is conditioned in this population by the judicial criterion of subjective incredibility (Law of Precedent) that establishes that the testimony of the complainant is not sufficient proof of incrimination if he/she has any interest in the cause beyond the legitimate conviction of the accused (e.g., economic benefit, resentment, revenge, existence of a previous relationship).

Regarding attributional biases, the results confirmed that, for judgment making and, by extension, judicial decision-making, judicial agents use sources of informal reasoning (attributional biases) in contrast to the formal reasoning expected in this context. These biases facilitate paths of judgment both incriminating (i.e., attribution of responsibility to the accused, attribution of credibility to the complainant, attribution to the facts of a nature of rape) and exculpatory (i.e., attribution of responsibility to the complainant, attribution of the duty of prevention to the complainant). Furthermore, the magnitude of the effects of the biases in the reasoning is more than great. However, the biases in favor of incriminating the accused (attribution of responsibility to the accused, attribution of credibility to the complainant, attribution to the facts a nature of rape) have a greater weight than those that delegitimize the accusation (attribution of responsibility to the complainant, attribution of the duty of prevention to the complainant). Moreover, the results revealed that these have a smaller effect on known victims than on unknown ones. Succinctly, higher effects for attributional biases are for unknown perpetrators in both directions: to support incrimination (i.e., attribution of higher veracity to complainant testimony) and absolution/dismiss of the judicial proceeding (i.e., higher attribution of responsivity to complainant, higher attribution of capacity to prevent the incident to complainant). Nevertheless, the attribution of credibility to the complainant testimony has a higher incriminating evidence value than the attribution of responsivity and capacity to prevent the incident to the complainant for exonerating criminal responsibility (Arce et al., 2003). In this way, the existence of a close link between the complainant and the accused requires a greater burden of proof, since less verisimilitude is attributed to the complainant testimony (McKimmie et al., 2014), more responsibility is attributed to the complainant in the facts and more ability to have prevented the incident (Hohl and Stanko, 2015; Hine and Murphy, 2017). In the absence of such an increased burden of proof, these attributional biases would predispose judgment making toward the absolution of the prosecuted or the dismiss of the judicial proceeding. Paradoxically, this is related to a lower probability of reporting and abandoning the relationship (Garrido-Macías et al., 2020) and, by extension, less judicial protection for victims of known aggressors. However, the case study warns that around 1/3 of the judicial agents (34.0, 30.2, and 28.8%, respectively), would attribute, respectively, more responsibility, truthfulness, and prevention capacity to the complainant when the perpetrator is known (statistical model error).

Motivational attribution biases refer to a tendency to form and hold beliefs that conform to the needs of the individual, in this case, judgment making and the subsequent decision-making, and manifest when the legal evidence is insufficient or weak (week cases; Butterfield and Bitter, 2019), being irrelevant in strong evidence cases (Visher, 1987; Kahneman, 2011). Under this contingency, judicial agents, in judicial judgment and decision making, must be guided by strict compliance with the principle of presumption of innocence (Article 11.1 of the Universal Declaration of Human Rights; United Nations, 1948), which implies that none innocent person may be classified as guilty, so the attributional biases supporting the absolution of the accused of the crime would support the motivation of the procedural action taken or the judicial resolution. However, although the judicial resolution or procedural action (i.e., prosecutorial decision making) executed would be correct, it would be based on informal reasoning, which would deviate from normative judicial reasoning (i.e., evidenceless, unfounded). On the contrary, in cases of weak or insufficient legal evidence (burden of proof), resorting to attributional biases to support the case would not only be inadmissible in terms of reasoning (motivation of the actions or judicial resolutions) as it is informal in the face of the expected formal, but also contrary to law (judicial error). In any case, attributional biases, such as irrational beliefs, provide such a high level of support (effect size greater than large) that they give the subject a guarantee of certainty and efficiency in judgment making (Perry, 1988).

In conclusion, although generally and in population terms the effect of biases in judgment making contrary to complainant is not observed, in the case study it was found that a large number of judicial agents participated of biased routes against the complainant. Moreover, this type of bias in judgment making does not occur in other crimes (Bieneck and Krahé, 2011). This research found that in rape cases was attributed more blame to the victim and less blame to the perpetrator compared with robbery cases. Thus, as these sources of bias in judgment making are unconscious for judgment makers and ways of informal reasoning, judicial agents should be trained to control the effects of these sources of bias (Bartels, 2010), promoting debiasing, i.e., substituting informal reasoning (judgment making sustained on biases against the complainant) by formal reasoning sources (evidence, procedural rules, charge of the proof) (Butterfield and Bitter, 2019). In sum, the training and specialization of judicial agents (e.g., courts specialized in sexual assaults, training police forces to obtain the statement from complainants of sexual assault) in sexual violence against women cases is necessary (Barn and Powers, 2021; Gancedo et al., 2021b); so that, from an orientation of Therapeutic Justice, they can mediate the wellbeing of the victims (Cattaneo and Goodman, 2010; Camplá et al., 2020; Novo et al., 2020).

## Limitations

The results of this study are not generalizable to other types of populations, since judicial agents are determined in their judgments by procedural and legal rules and Chilean Law of Precedent. Likewise, caution must be taken in generalizing judicial agents from judicial contexts other than Chile, since the case law may not be equivalent. As participation was voluntary, the results do not represent strictly the population. Finally, the manifestation of these biases can only be generalized to cases of insufficient evidence.

## Data availability statement

The raw data supporting the conclusions of this article will be made available by the authors, without undue reservation.

## Ethics statement

Ethical review and approval was not required for the study on human participants in accordance with the local legislation and institutional requirements. The patients/participants provided their written informed consent to participate in this study.

## Author contributions

All authors listed have made a substantial, direct, and intellectual contribution to the work, and approved it for publication.
